# Structural Characterization, Antioxidant and Antibacterial Activities of a Novel Polysaccharide From *Zingiber officinale* and Its Application in Synthesis of Silver Nanoparticles

**DOI:** 10.3389/fnut.2022.917094

**Published:** 2022-06-03

**Authors:** Yongshuai Jing, Wenjing Cheng, Yunfeng Ma, Yameng Zhang, Mingsong Li, Yuguang Zheng, Danshen Zhang, Lanfang Wu

**Affiliations:** ^1^College of Chemistry and Pharmaceutical Engineering, Hebei University of Science and Technology, Shijiazhuang, China; ^2^College of Pharmacy, Hebei University of Chinese Medicine, Shijiazhuang, China

**Keywords:** *Zingiber officinale* (ginger), polysaccharides, nanoparticles, nano-silver, antioxidant activity, antibacterial activity

## Abstract

A novel polysaccharide (ZOP) was extracted from *Zingiber officinale* with ultrasonic assisted extraction method. ZOP monosaccharide composition and mole ratio is GlcA: GalA: Glc: Gal: Ara = 1.97:1.15:94.33:1.48:1.07. Then, the particle size of ZOP-NPs prepared by nano-precipitation method was 230.5 nm, and the polydispersity index (PDI) was 0.260. Using ZOP and ZOP-NPs as reductants and stabilizers, ZOP-AgNPs and ZOP-NPs-AgNPs were prepared. They were characterized by ultraviolet-visible spectrophotometer (UV-Vis), fourier transform infrared spectroscopy (FT-IR), scanning electron microscope (SEM), transmission electron microscope (TEM), and X-ray diffraction (XRD). The silver chelation rate of polysaccharide silver nanoparticles (AgNPs) ranged from 68.70 to 82.12%. ZOP-AgNPs (0.5%, w/v; 1%, w/v) and ZOP-NPs-AgNPs (0.5%, w/v; 1%, w/v) exhibited a narrow particle size distribution of 31.1, 34.6, 25.1 and 27.6 nm, respectively. And the zeta potential values of them were−19.4,−21.6,−19.7,−23.8mV, respectively. The antioxidant and antibacterial activities of ZOP-NPs-AgNPs were superior to those of ZOP, ZOP-NPs and ZOP-AgNPs.

## Introduction

In this millennium, nanotechnology, as an advanced material science, has gradually entered the field of medicine. Nanoparticles are said to be raw materials used in nanotechnology (1). These raw materials are found in different types, e.g., gold, copper, iron, nickel, and silver nanoparticles (2). Polysaccharide nano-silver (AgNPs) has attracted wide attention due to its high antibacterial, anti-inflammatory and anticancer activities, and is widely used in personal care products, textiles, food packaging, building materials and medical devices (3). The preparation methods of nano-silver include physical method, chemical reduction method and biological reduction method (4). Because most of the physical and chemical reduction methods are high energy consumption, high cost, toxic, non-environmental protection and low productivity, today's nanotechnology needs to adopt various green biological reduction methods to synthesize nanoparticles (5). Polysaccharides can be used as natural reducing agents because of their excellent biocompatibility, wide sources, biodegradability and non-toxicity. At the same time, no toxic substances are produced in the synthesis process, and the whole synthesis process is very green and environmentally friendly. The nano-silver particles generated by polysaccharide have the advantages of high stability and difficult agglomeration, and the addition of polysaccharide gives the nano-silver some other biological activities (6).

*Zingiber officinale* is a plant belonging to the Zingiber aceae family, contains polyphenols, terpenes, polysaccharides, gingerol and its derivatives and other active ingredients. It has certain therapeutic effects on inflammation, high cholesterol, tumor, atherosclerosis, ulcers, dyspepsia, diabetes, cardiovascular diseases and so on (7, 8). With the increase of *Z. officinale* production, it can be processed into many other foods, such as condiments, syrups, candy, dry powder and so on (9). At the same time, there will be more and more by-products, namely *Z. officinale* pomace. *Z. officinale* pomace is also rich in bioactive components, especially polysaccharides. A lot of data suggested that *Z. officinale* polysaccharide has broad application prospect in disease prevention and health care product development, and has the advantages of anti-tumor, anti-oxidation, hypoglycemic, antitussive, anti-fatigue, high safety and low toxic and side effects (10, 11).

Considering the ideal properties of ZOP and silver nanoparticles, polysaccharides from *Z. officinale* pomace were extracted, nanoparticles and silver nanoparticles with different concentrations or different UV irradiation time were prepared. Their physical and chemical properties such as UV-Vis, FT-IR, SEM, TEM, and XRD were studied, and their antioxidant and antibacterial activities were compared. This study provides a green method for preparing nano-silver. At the same time, it is hoped that the pomace of traditional Chinese medicine can be better utilized in the future.

## Materials and Methods

### Experimental Materials and Reagents

*Z. officinale* (No. 2019031401) was purchased from AnguoYaoyuan Trading Co., LTD. The sample was identified as the rhizome of *Zingiber officinale* Roscoe by associate professor Lanfang Wu (Department of Pharmacy, Hebei University of Chinese Medicine). A voucher specimen was deposited at the College of Chemistry and Pharmaceutical Engineering, Hebei University of Science and Technology, China. Standard monosaccharides, T-series dextrans, dimethyl sulfoxide (DMSO), 1-phenyl-3-methyl-5-pyrazolone (PMP), 1,1-diphenyl-2-picrylhydrazyl (DPPH), NaCl, NH_3_·H_2_O and AgNO_3_ were purchased from Shanghai Aladdin Biochemical Technology Co., Ltd. *Escherichia coli* (*E. coli*, ATCC 8739) was used in this experiment. All reagents were analytical grade.

### Extraction of ZOP

The rhizome of *Z. officinale* was crushed, and its powder (100 g) was extracted with 1:13 ethanol reflux for twice in 2 h to remove pigment and lipid, and remove supernatant. The dried ginger residue after fat removal and distilled water (1:30 w/v) were placed in a round-bottom flask, and ginger polysaccharide was extracted by ultrasonic assisted extraction. The specific conditions were as follows: ultrasonic extraction for 20min, then boiling water extraction (90°C), twice in 2 h. The supernatant was concentrated with 80% ethanol, precipitated at 4°C for 12 h, centrifuged (3,000×g, 10min, 4°C), and lyophilized to obtain the precipitate (named ZOP).

### Physicochemical Properties of ZOP

#### Determination of Total Soluble Sugar Content

The total soluble sugar content in ZOP was determined by phenol-sulfuric acid method (12).

#### Determination of Average Molecular Weight

The molecular weight of ZOP was determined by high performance gel permeation chromatography (HPGPC) (13). It was equipped with TSK-GEL G5000PW_XL_ (300mm × 7.8mm, i.d.) and G3000PW_XL_ (300mm × 7.8mm, i.d.) gel columns in series (Tosoh Biosep, Japan) and a Waters 2414 refractive index detector (Massachusetts, USA). The samples were eluted at 0.6 mL/min flow rate with monopotassium phosphate solution as mobile phase. The T-series dextran was used as standard.

#### Determination of Monosaccharide Composition

The monosaccharide composition of ZOP was analyzed by HPLC with pre-column derivatization (14). In simple terms, 2 mol/L trifluoroacetic acid and ZOP were hydrolyzed in a sealed tube at 110°C for 4 h, followed by PMP derivatization. The monosaccharide composition analysis was performed on an ODS C18 column (4.6mm × 250mm) connected to an Agilent 1260 HPLC system. The standard monosaccharides were determined under the same conditions described above (15).

### Preparation of Silver Nanoparticles From Polysaccharides

**Figure 1:**
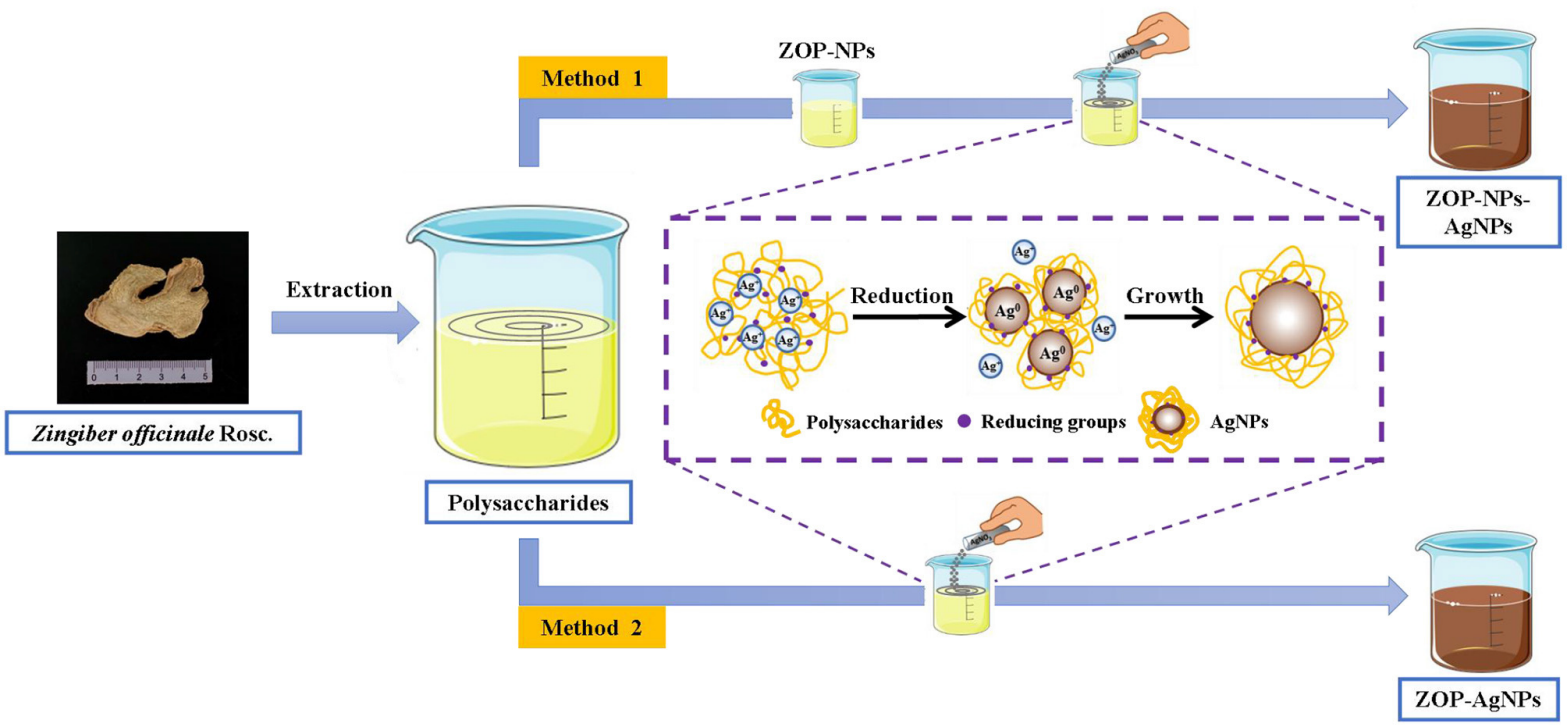
Schematic representation of ZOP-NPs, ZOP-AgNPs, ZOP-NPs-AgNPs synthesis.

The green methods for silver nanoparticles synthesis using ZOP and ZOP-NPs were as Figure 1.

#### Preparation of ZOP-NPs

0.5 g and 1 g ZOP were dissolved in 100mL deionized water to prepare 0.5% (w/v) and 1% (w/v) polysaccharide solutions, respectively. The polysaccharide solution was then added to methanol in a ratio of 1:10 and stirred magnetically. All polysaccharide solutions were dropped into methanol and stirred mechanically for 2 h to obtain nano-polysaccharide solutions. ZOP-NPs was then obtained by freeze-drying (16).

#### Preparation of AgNPs

ZOP-AgNPs and ZOP-NPs-AgNPs were prepared under the condition that the solution volume ratio of polysaccharide (0.5%, w/v; 1%, w/v), AgNO_3_ (0.05 mol/L) and NaCl (1 mg/L) was 1: 2: 1.5. Adjust the mixture to pH = 8.7 with NH_3_·H_2_O. Subsequently, the mixture was placed in a magnetic agitator and reacted for 4 h at room temperature with 365 nm UV irradiation and continuous agitation to form a brown AgNPs solution. After the reaction, the precipitate was collected by repeated centrifugation (9,000 × g ; 15min) and lyophilized (17).

Volhard method was used to determine the chelation rate of silver ions in polysaccharide nanoparticles. In this method, ferric ammonium alum is used as a tracer agent and ammonium thiocyanate as a standard solution to titrate silver ions in solution.


$$\begin{array}{l} A\left(\%\right)=\frac{C_{0}-C}{C_{0}}\times 100\% \end{array}$$

where, A, C_0_ and C were the chelation rate (%), the concentration of silver ion in the solution before the reaction (mg/mL) and the concentration of silver ion in the solution after the reaction (mg/mL).

#### Size and Polydispersity Indexanalysis

Appropriate amount of AgNPs prepared above was diluted with ultra-pure water (0.1%). The particle size, polydispersity index (PDI) and Zeta potential were determined by using Zetasizer Nano dynamic light scattering (DLS) technique.

### Characterization of AgNPs

#### Thermal Analysis

The thermal stability of the samples was determined by TGA-DSC (TA Instruments Ltd., Q600, America). N_2_ was used as a protective gas to heat the sample from room temperature to 800°C at a temperature of 10°C/min.

#### UV-Vis Spectroscopy Analysis

The AgNPs obtained by the reaction was scanned in the range of 200–800 nm with UV-vis Spectrophotometer (18).

#### FT-IR Spectroscopy Analysis

Infrared absorption spectra of ZOP, ZOP-NPs, ZOP-AgNPs, and ZOP-NPs-AgNPs were recorder by S-100 FT-IR Spectrometer (PerkinElmer, America) at the frequency of 4,000–400 cm^−1^ range.

#### SEM Analysis

ZOP, ZOP-NPs, ZOP-AgNPs, and ZOP-NPs-AgNPs after gold spraying were analyzed by SEM (JSM 7610F, JEOL Ltd. Japan). The acceleration voltage was 3.0 kV and the resolution was 7.1mm. The micro solid morphology of ZOP, ZOP-NPs, ZOP-AgNPs, and ZOP-NPs-AgNPs were photographed and recorded at different magnification and the element distributions of them were determined by SEM-EDS-mapping.

#### TEM Analysis

The synthesized nano silver sol sample was directly used for TEM testing. The carbon supporting film carrying the sample was placed on a clean filter paper, a small amount of sample liquid was absorbed with a pipette gun, a drop of sample liquid was dropped on the carbon supporting film, and then placed in a 40°C oven to dry or air dry at room temperature. The preparation of samples to be tested was completed. Then the samples were analyzed by TEM JM-2100.

#### XRD Analysis

The crystal structure of the samples was analyzed by X-ray diffractometer model (XRD-6000, Shimadzu, Japan). Cu-Kα ray (λ = 0.15406 nm) was used as the target, the tube voltage was 40 kV, the tube current was 30mA, the scanning rate was 5 °/min, and the diffraction Angle was 2θ = 10–90° (19).

#### Antioxidant Activities

The scavenging activities of ZOP, ZOP-NPs, ZOP-AgNPs, and ZOP-NPs-AgNPs against DPPH· and OH· were determined at different concentrations (20).

#### Antibacterial Activity

The antibacterial activity of the samples against *E. coli* was investigated by oxford cup method. The agar medium was prepared by inverted dish method, sterilized, cured and inoculated with bacterial suspension. Different concentrations of Ag nanoparticles, nano-polysaccharide solutions and ZOP solutions were placed in precultured agar mediums. The mediums were incubated at 37°C for 24 h. Then the diameter was measured (21).

#### Data Statistics and Analysis

Microsoft Excel 2019 and SPSS 26.0 were used to process and analyze the data, and Origin 2019 was used for drawing.

## Results and Discussion

### Physicochemical Properties of ZOP

#### Determination of Total Soluble Sugar Content

The crude polysaccharide of *Z. officinale* was 6.77 g by ultrasonic assisted boiling water extraction and ethanol precipitation. The glucose standard curve equation for determination of *Z. officinale* polysaccharide content by phenol-sulfuric acid method was: *y* = 15.673*x*-0.0153 (*R*^2^ = 0.9996), and the linear range was 0–0.1 mg/mL. Therefore, the total soluble sugar content of ZOP was (78.6 ± 0.6)%.

#### Determination of Average Molecular Weight

**Figure 2:**
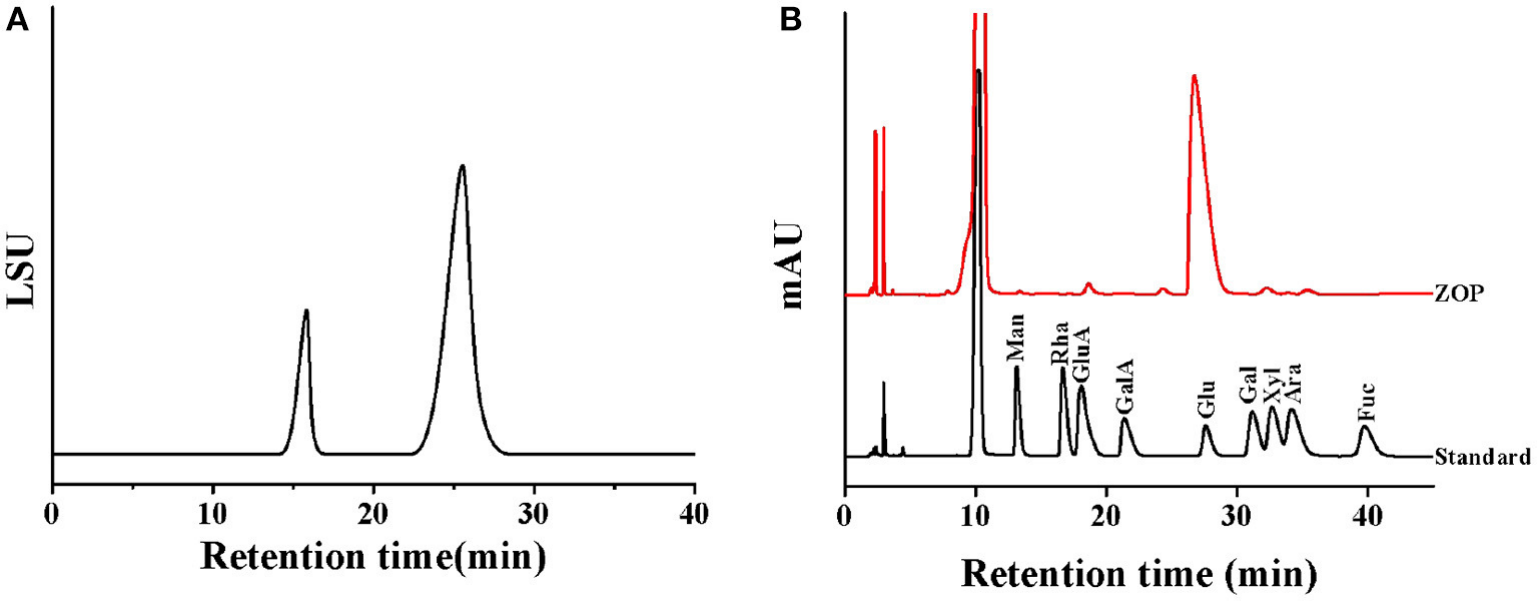
The physical and chemical properties analysis of ZOP. Molecular weight distribution of ZOP **(A)**, HPLC of standard monosaccharides and ZOP **(B)**.

The high performance gel permeation chromatogram of ZOP was shown in Figure 2A. The regression equation of the standard curve of dextran molecular weight was *y*=−0.3108*x*+11.749 (*R*^2^ = 0.9911), and the molecular weight of two different components of ZOP were 6.04 × 10^6^ Da (7.17%) and 5.42 × 10^3^ Da (92.83%), respectively.

#### Determination of Monosaccharide Composition

After HPLC analysis, the monosaccharide composition of ZOP was determined by comparing the retention time with that of standard monosaccharide (Figure 2B). The results showed that the monosaccharide composition and molar ratio of ZOP were GlcA: GalA: Glc: Gal: Ara = 1.97:1.15:94.33:1.48:1.07.

### AgNPs Characterization

#### Silver Chelation Rate and Elemental Analysis

**Figure 3:**
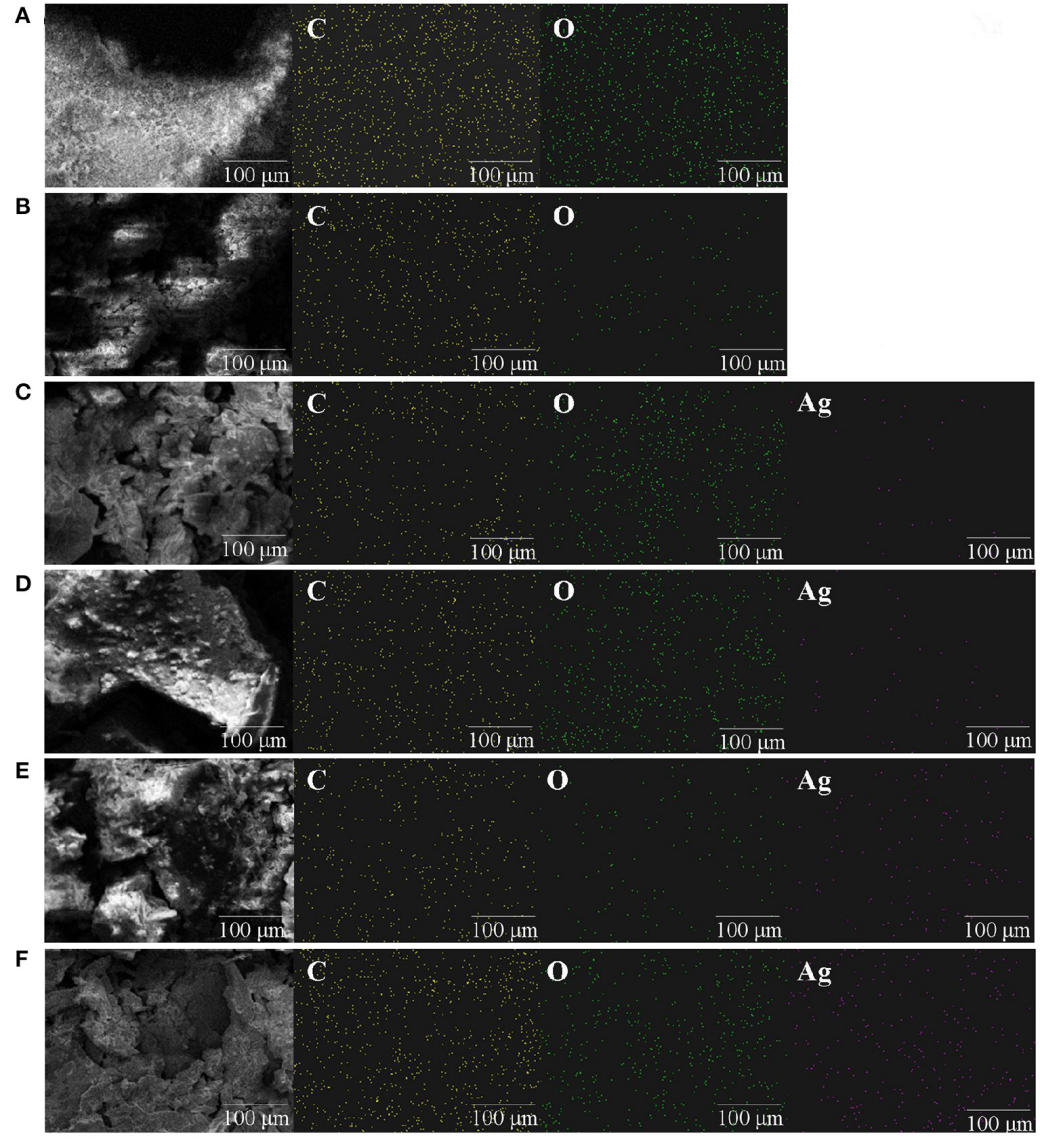
Mapping images of ZOP **(A)**, ZOP-NPs **(B)**, 0.5% (w/v)-ZOP-AgNPs **(C)**, 0.5% (w/v)-ZOP-NPs-AgNPs **(D)**, 1% (w/v)-ZOP-AgNPs **(E)**, 1% (w/v)-ZOP-NPs-AgNPs **(F)** ( ×30,000).

Polysaccharide, as a reducing agent and stabilizer, plays an important role in the synthesis of polysaccharide silver nanoparticles (22). The silver chelating rates of 0.5% (w/v)-ZOP-AgNPs, 0.5% (w/v)-ZOP-NPs-AgNPs, 1% (w/v)-ZOP-AgNPs, and 1% (w/v)-ZOP-NPs-AgNPs were 68.70, 72.28, 79.88, and 82.12%, respectively. In addition, when the reaction time, solution volume ratio and AgNO_3_ concentration were the same, the chelating rate of AgNPs increased with the increase of polysaccharide concentration. After comparison, it was found that the ZOP-NPs-AgNPs prepared by ZOP-NPs had a higher silver chelation rate. Moreover, according to the analysis of the Mapping spectrum (Figure 3), the silver content in the AgNPs was positively correlated with the polysaccharide concentration, and the silver content in the AgNPs after the polysaccharide nanocrystallization was higher, which was consistent with the results of the determination of silver chelation rate.

#### Particle Size and Zeta Potential Analysis

The results were as follows: the particle size of ZOP-NPs was 230.5 nm, and the PDI value was 0.260, the ZOP-AgNPs (0.5%, w/v; 1%, w/v) and ZOP-NPs-AgNPs (0.5%, w/v; 1%, w/v) exhibited a narrow particle size distribution of 31.1, 34.6, 25.1 and 27.6 nm, respectively. In addition, their PDI values were 0.394, 0.431, 0.368, 0.387, respectively.

The larger the PDI, the wider the molecular weight distribution; The smaller the PDI, the more uniform the molecular weight distribution (23). After comparison, it was found that ZOP-AgNPs and ZOP-NPs-AgNPs particle size and PDI were positively correlated with polysaccharide concentration. Under the condition of the same polysaccharide concentration, the particle size and PDI of ZOP-NPs-AgNPs was smaller, and 0.5%-ZOP-NPs-AgNPs had the smallest particle size and PDI, and the best dispersion. The colloidal solution of nanoparticles had a larger negative potential, so it had a higher electrostatic stability. The zeta potential values of ZOP-AgNPs (0.5%, w/v; 1%, w/v) and ZOP-NPs-AgNPs (0.5%, w/v; 1%, w/v) were −19.4, −21.6, −19.7, −23.8 mV, respectively (Table 1). Their zeta potential values were larger, which confirms that the nano-silver synthesized by ZOP in this experiment had good stability. After comparison, it was found that 1%-ZOP-NPs-AgNPs had higher stability.

**Table 1:** Zeta potential, particle size and distribution of ZOP, ZOP-NPs, ZOP-AgNPs, and ZOP-NPs-AgNPs.

**Name**	**Size/nm**	**PDI**	**Zeta potential/mV**
ZOP	345.2	0.520	−10.9
ZOP-NPs	230.5	0.260	−27.1
0.5% (w/v)-ZOP-AgNPs	31.1	0.394	−19.4
0.5% (w/v)-ZOP-NPs-AgNPs	25.1	0.368	−19.7
1% (w/v)-ZOP-AgNPs	34.6	0.431	−21.6
1% (w/v)-ZOP-NPs-AgNPs	27.6	0.387	−23.8

#### TGA-DSC Analysis

**Figure 4:**
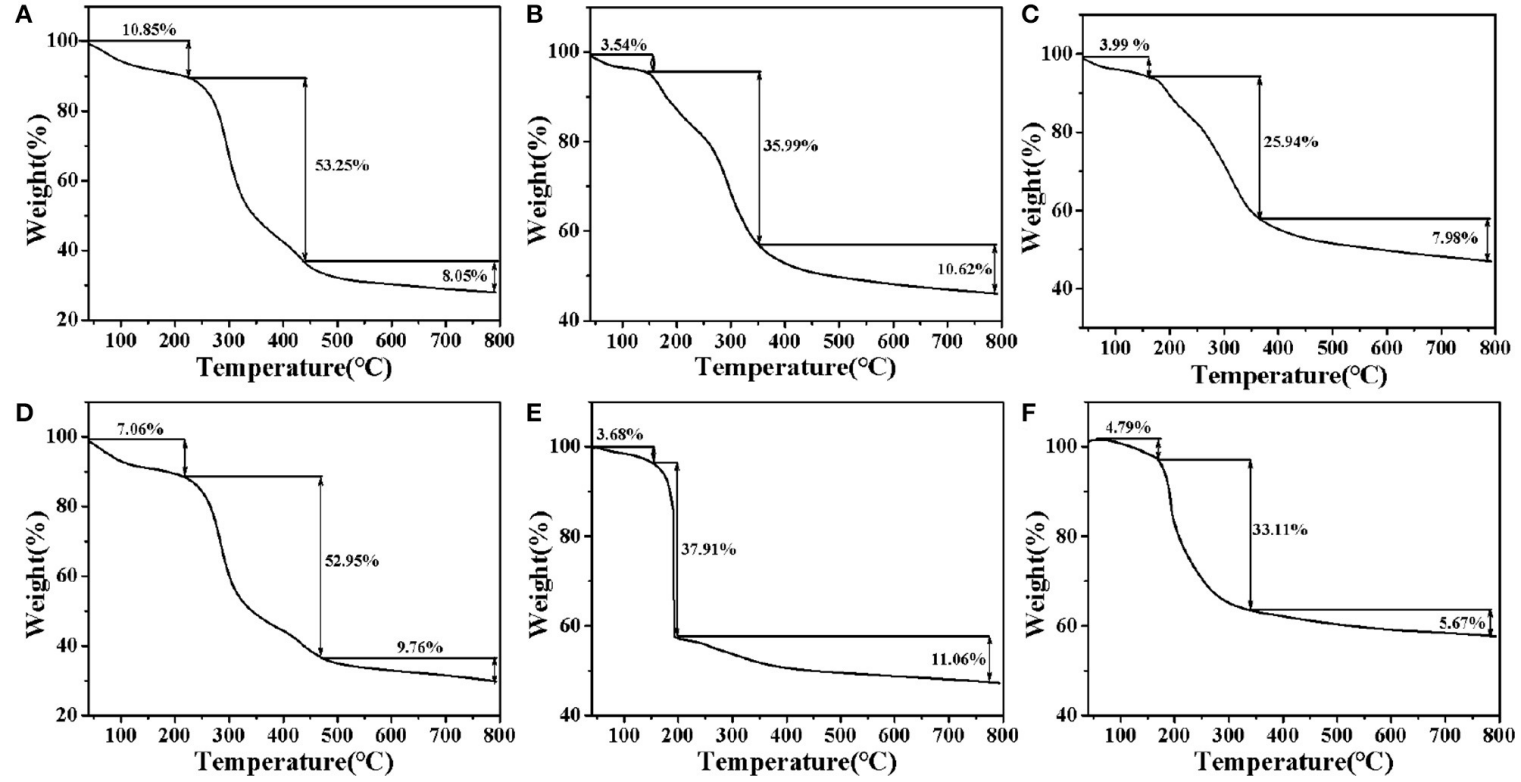
DSC-TGA analysis of ZOP **(A)**, 0.5% (w/v)-ZOP-AgNPs **(B)**, 1% (w/v)-ZOP-AgNPs **(C)**, ZOP-NPs **(D)**, 0.5% (w/v)-ZOP-NPs-AgNPs **(E)**, and 1% (w/v)-ZOP-NPs-AgNPs **(F)**.

The changes of ZOP, ZOP-NPs, ZOP-AgNPs, and ZOP-NPs-AgNPs with temperature were shown in Figure 4. Due to loose binding water in the polysaccharide, the mass of ZOP decreased slightly between 0 and 210°C, and the weight loss rate of ZOP was 16.8%. When the temperature was about 220°C, the thermal decomposition of polysaccharide itself occurred, leading to the breaking of glycosidic bonds and ring-opening reaction. The weight of ZOP decreased sharply by 63.25% between 220 and 800°C (24).

It can be seen from Figure 4D that the thermal degradation process of ZOP-NPs did not changed. From the initial degradation temperature, it can be seen that the thermal stability of ZOP and ZOP-AgNPs were similar. Thermal decomposition curve of ZOP-AgNPs (0.5%, w/v; 1%, w/v), as shown in Figures 4B,C. ZOP-AgNPs (0.5%, w/v; 1%, w/v) weightlessness in the process of 25–170°C was mainly caused by water loss. Their weight loss rates are 3.54 and 3.99%, respectively. The second and third stages were 170–370°C and 370–800°C, respectively. In these two stages, the weight loss rates of 0.5%-ZOP-AgNPs and 1%-ZOP-AgNPs were 46.61 and 33.92%, respectively. Figures 4E,F illustrates the thermogravimetric analysis of ZOP-NPs-AgNPs (0.5%, w/v; 1%, w/v). ZOP-NPs-AgNPs (0.5%, w/v; 1%, w/v) of the first stage of weightlessness rate were 3.68 and 4.79%, respectively. The second stage and third stage of 0.5%-ZOP-NPs-AgNPs were 160–190°C and 190–800°C, respectively. And the weight loss rate of these two stages was 48.97%. The second and third stages of 1%-ZOP-NPs-AgNPs were 160–340°C and 340–800°C, respectively, and the weight loss rate of these two stages was 38.78%. After comparison, it was found that the weight loss rate of ZOP-NPs-AgNPs was slightly higher than that of ZOP-AgNPs, which might because of the lesser particle size of ZOP-NP-AgNPs and easier to decompose. It can be seen that water loss and thermal decomposition of polysaccharide itself are the main causes of thermal weight loss of nano-silver polysaccharide. In addition, the weight loss rate of the modified silver nanoparticles decreased significantly, and with the increase of silver concentration in the AgNPs, the weight loss rate of the AgNPs also decreased significantly, indicating that the AgNPs could protect and stabilize the silver nanoparticles in the synthesis process (25).

#### UV-Vis Spectroscopy Analysis

**Figure 5:**
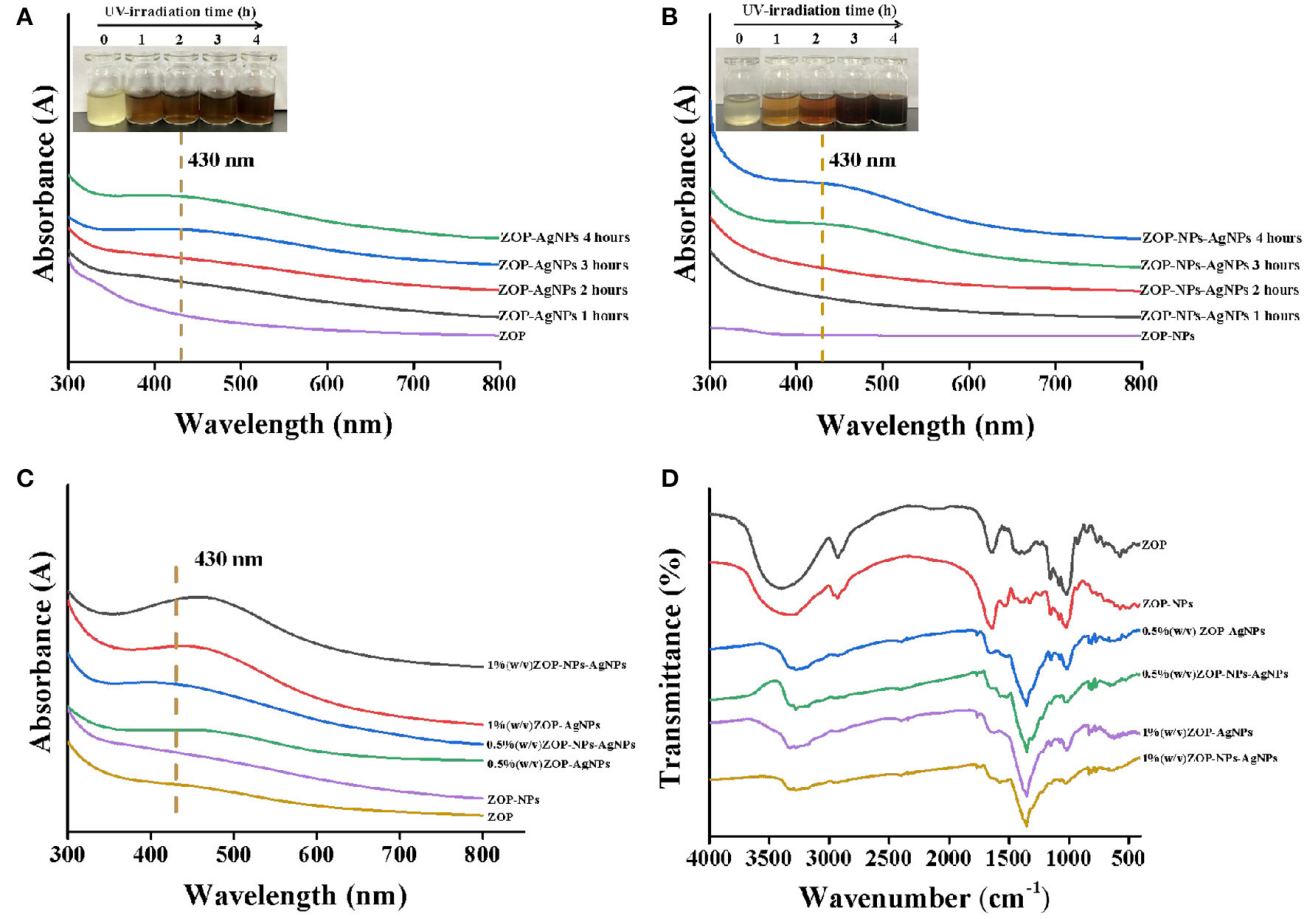
UV-vis of ZOP-AgNPs **(A)** and ZOP-NPs-AgNPs **(B)**under different time conditions; UV-Vis **(C)** and FT-IR **(D)** analysis of ZOP, ZOP-NPs, ZOP-AgNPs, and ZOP-NPs-AgNPs.

UV-Vis spectroscopy was an important technique to determine the structure of polysaccharides and the formation of metal nanoparticles. It could be seen from Figures 5A,B that there were no absorbances peak at 280 and 260 nm in UV-Vis, indicating that ZOP and ZOP-NPs does not contain protein and nucleic acid. With the increase of UV-irradiation time, the color of the prepared ZOP-AgNPs and ZOP-NPs-AgNPs solutions changed from light yellow to yellowish brown. This was due to silver nanoparticles absorbed the radiation in the visible region of electromagnetic spectrum, resulting in the reaction mixture becoming a dark brown solution (26). In the UV absorption spectrum, the wider absorption peak at 430 nm was typical surface plasmon resonance absorption of silver nanoparticles, which also indicated the formation of spherical silver nanoparticles. As can be seen from Figure 5C, ZOP-NPs-AgNPs showed stronger absorption peaks than ZOP-AgNPs. Moreover, the absorption peak was positively correlated with the polysaccharide concentration. This indicated that when the polysaccharide concentration sincreased, more polysaccharides participate in the reduction of silver, and more AgNPs was generated. At the same time, there were no other peaks in the range of 300–600 nm, indicating that there was no aggregation or cluster formation of silver particles (27).

#### FT-IR Analysis

ZOP, ZOP-NPs, ZOP-AgNPs, and ZOP-NPs-AgNPs were characterized by FT-IR spectra, and the results were shown in Figure 5D. In the FT-IR spectrum of ZOP (Figure 5D), a broad peak around 3,400 cm^−1^ is the characteristic of O-H stretching frequency which shifts to 3,250 cm^−1^ and becomes narrower for ZOP-AgNPs. The absorption peak at 2,929 cm^−1^ was due to -CH_2_ stretching vibration. A band at 1,642 cm^−1^ was attributed to stretching vibration of C=O group of ester group. The absorption peak at 1,416 and 1,370 cm^−1^ were observed due to the C-H bending vibrations. The absorption peak at 1,155, 1,081, and 1,021 cm^−1^ were due to the stretching variation of C-O-C group (28). In case of AgNPs the peaks corresponding to the above functionalities appeared at 2,931, 1,644, 1,350, and 1,151 cm^−1^, respectively. The abundant hydroxyl, carboxyl and macromolecular matrix structures on the surface of natural polysaccharides endows polysaccharides with the ability to stabilize AgNPs (29). It can be seen from Figure 5D that compared with ZOP and ZOP-NPs, the band shift in O-H and the increased band intensities for C=O in the FT-IR spectra of AgNPs prepared by polysaccharides increased, which can be inferred that both hydroxyl and carbonyl groups of ZOP and ZOP-NPs are involved in the synthesis of silver nanoparticles. Figure 5D also shown that the infrared visible spectrum of 1% (w/v)-ZOP-NPs and 1% (w/v)-ZOP-NPs-AgNPs were almost the same as that of 0.5% (w/v)-ZOP-NPs and 0.5% (w/v)-ZOP-NPs-AgNPs, except that 1% (w/v)-ZOP-NPs and 1% (w/v)-ZOP-NPs-AgNPs had a slight red shift (30).

#### SEM Analysis

**Figure 6:**
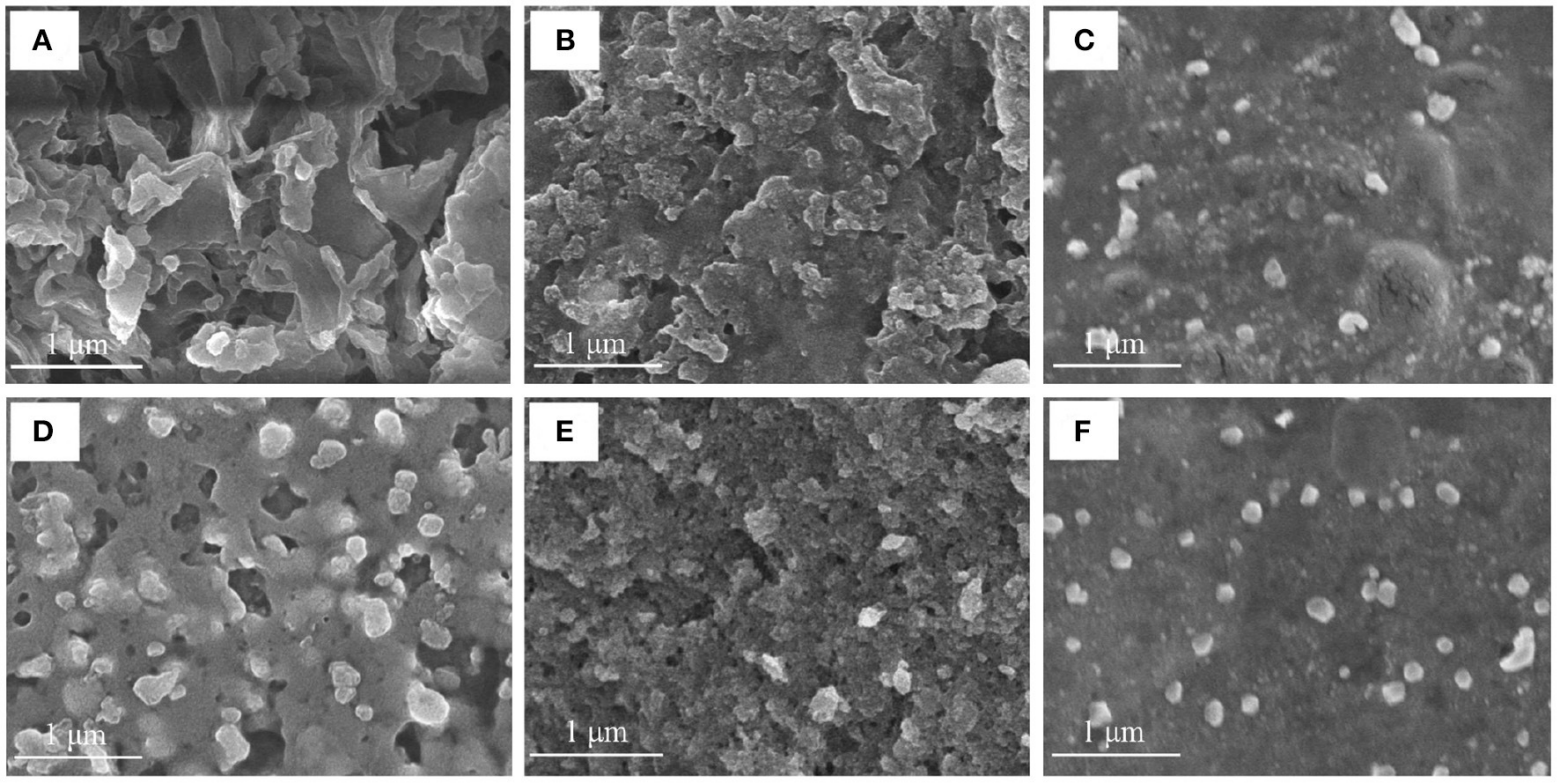
The SEM images of ZOP **(A)**, 0.5% (w/v)-ZOP-AgNPs **(B)**, 0.5% (w/v)-ZOP-NPs-AgNPs **(C)**, ZOP-NPs **(D)**, 1% (w/v)-ZOP-AgNPs **(E)** and 1% (w/v)-ZOP-NPs-AgNPs **(F)** ( ×30,000).

SEM of ZOP (Figure 6) shown that ZOP was a compact and stable sheet structure with smooth surface and irregular folds. Most of the shapes of the synthesized AgNPs were similar to spherical, while a few are lumpy, which might be caused by the fact that the synthesized AgNPs were frozen together in the freeze-drying process. Moreover, the particle size of AgNPs prepared by ZOP-NPs is smaller and the shape is more uniform.

#### TEM Analysis

**Figure 7:**
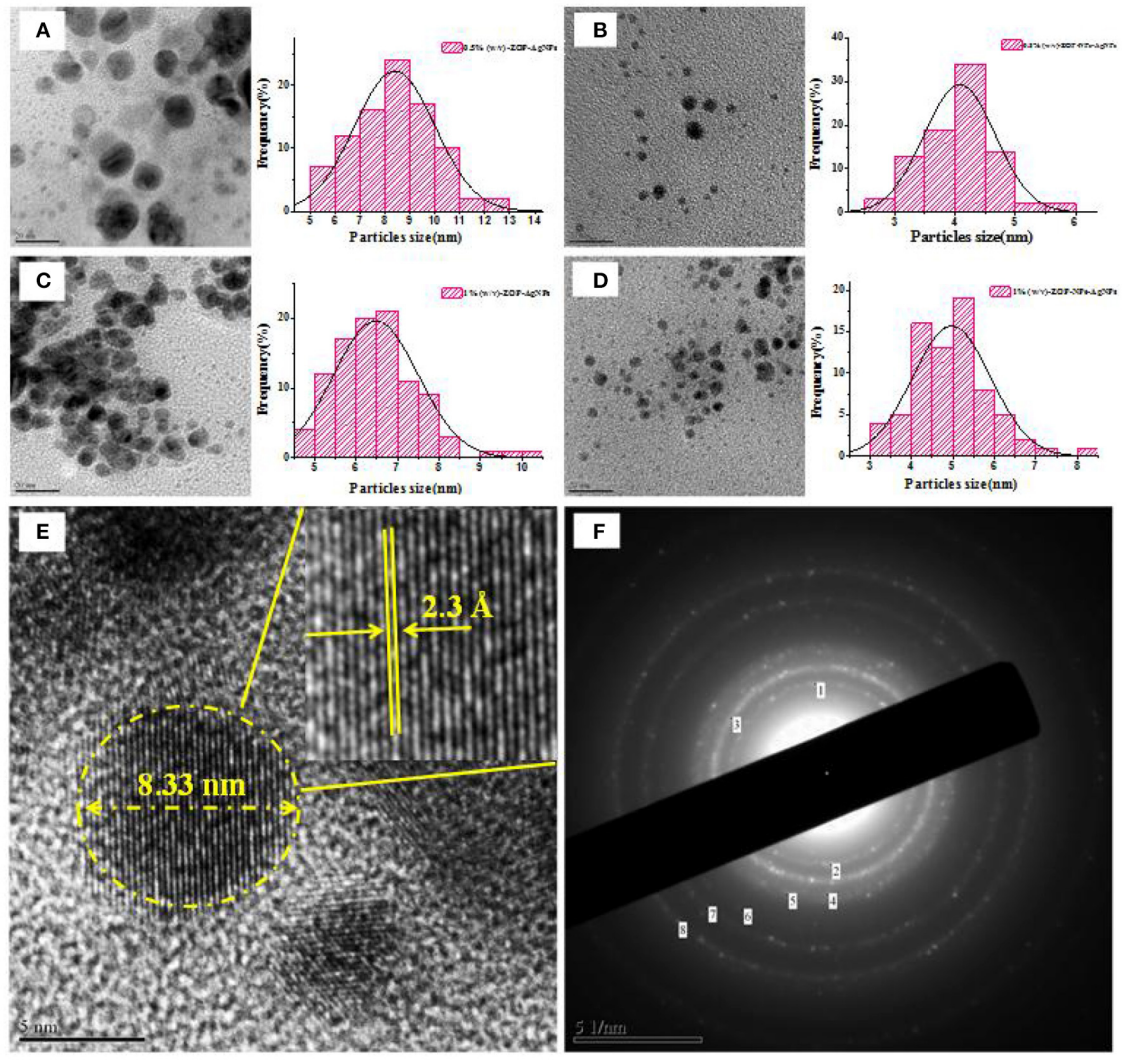
The TEM images of AgNPs and corresponding size distribution curve of AgNPs [**(A)** 0.5% (w/v)-ZOP-AgNPs; **(B)** 0.5% (w/v)-ZOP-NPs-AgNPs; **(C)** 1% (w/v)-ZOP-AgNPs; **(D)** 1% (w/v)-ZOP-NPs-AgNPs]; The TEM images of ultra-magnified image showing lattice fringe **(E)** and electron diffractogram of AgNPs **(F)**.

It could be observed from Figures 7A–D, ZOP-AgNPs and ZOP-NP-AgNPs were spherical with uniform distribution and few signs of aggregation, with particle sizes ranging from 2.89 to 8.14 nm. Further high resolution images confirmed that the ZOP-AgNPs and ZOP-NPs-AgNPs were highly crystalline in nature (Figure 7E). The lattice fringes of AgNPs could be observed in Figure 7E, and the distance was 2.11 Å, which supported the XRD data of AgNPs (the lattice plane is 111) (31). The crystal structure of AgNPs is also demonstrated by the selected area electron diffraction (SAED) mode (Figure 7F).

#### XRD Analysis

**Figure 8:**
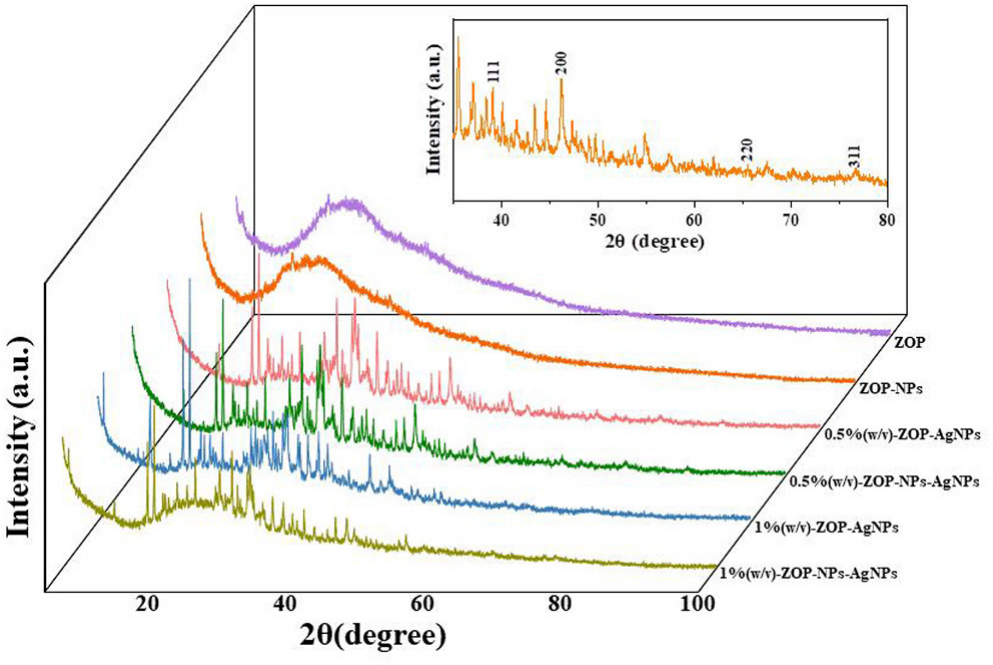
XRD analysis of ZOP, ZOP-NPs, 0.5% (w/v)-ZOP-AgNPs, 0.5% (w/v)-ZOP-NPs-AgNPs, 1% (w/v)-ZOP-AgNPs, 1% (w/v)-ZOP-NPs-AgNPs.

As shown in Figure 8, the XRD patterns of ZOP and ZOP-NPs show a low diffraction maximum at 10 ~ 30°, indicating that the internal structure of ZOP and ZOP-NPs was amorphous or semi-crystalline. XRD patterns of ZOP-AgNPs and ZOP-NPs-AgNPs showed four obvious diffraction peaks, which were located at (2θ) 38.28, 47.56, 64.56, and 77.52°, respectively, corresponding to the four planes of (111), (200), (220), and (311) of face-centered cube (FCC), which further confirmed the successful preparation of silver particles (30).

### Antioxidant Activities

**Figure 9:**
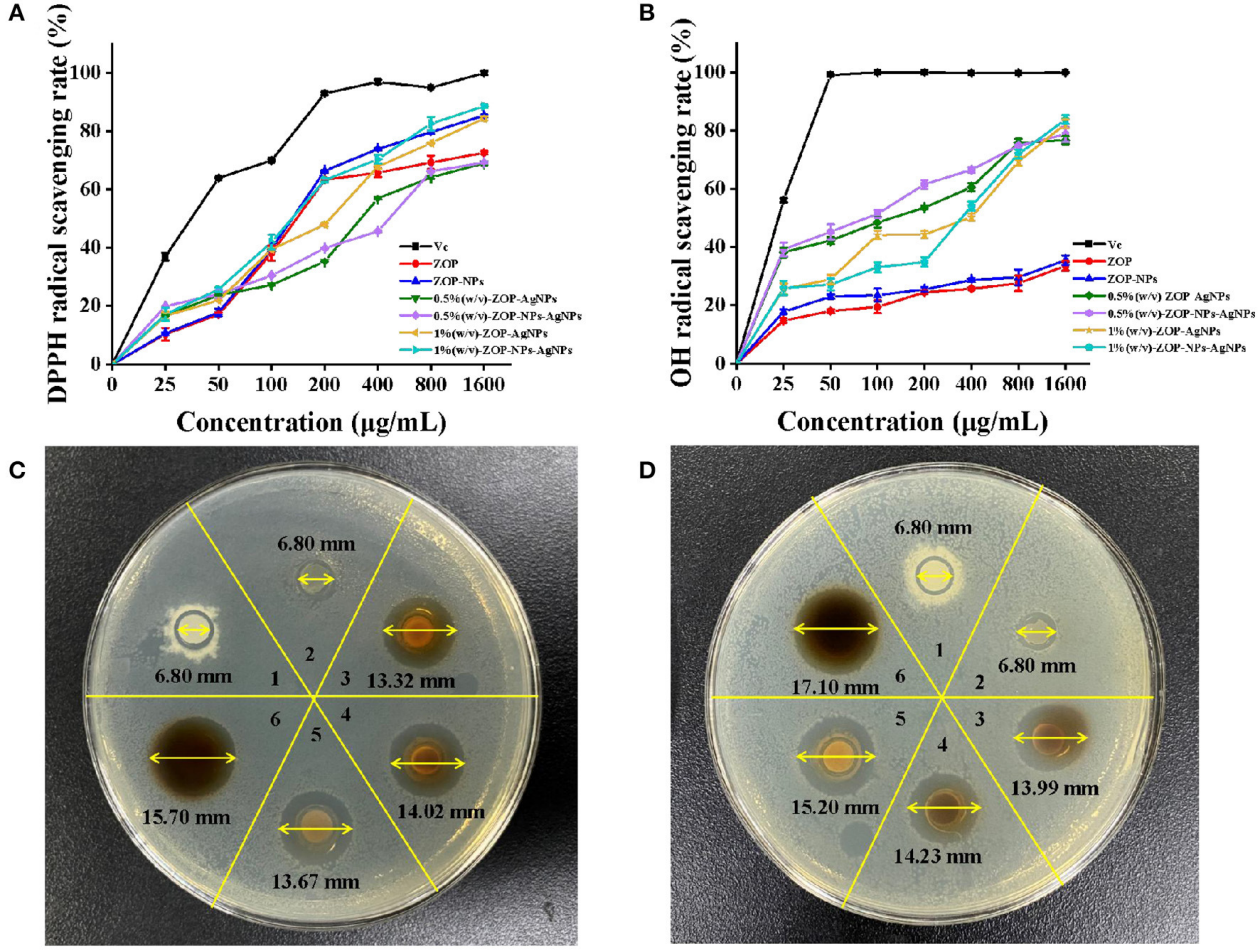
Antioxidant activities of ZOP, ZOP-NPs, ZOP-AgNPs, ZOP-NPs-AgNPs [**(A)** DPPH· radical; **(B)** OH· radical]; Antibacterial activity of ZOP, ZOP-NPs, ZOP-AgNPs, ZOP-NPs-AgNPs [1: ZOP; 2: ZOP-NPs; 3: 0.5% (w/v)-ZOP-AgNPs; 4: 0.5% (w/v)-ZOP-NPs-AgNPs; 5: 1% (w/v)-ZOP-AgNPs; 6: 1% (w/v)-ZOP-NPs-AgNPs; **(C)** 20 mg/mL; **(D)** 40 mg/mL].

Antioxidants are a class of substances that can help capture and neutralize free radicals, thereby reducing the risk of other degenerative diseases caused by reactive oxygen species (32). In this study, the ability of vitamin C (Vc), ZOP, ZOP-NPs, ZOP-AgNPs (0.5%, w/v; 1%, w/v), and ZOP-NPs-AgNPs (0.5%, w/v; 1%, w/v) to scavenge DPPH· and hydroxyl radicals were determined (Figures 9A,B). The half maximal inhibitory concentration (IC_50_) of ZOP, ZOP-NPs, 0.5% (w/v)-ZOP-AgNPs, 0.5% (w/v)-ZOP-NPs-AgNPs, 1% (w/v)-ZOP-AgNPs, 1% (w/v)-ZOP-NPs-AgNPs and Vc against DPPH· were 226.8, 164.3, 370.2, 376.9, 192.2, 141.6, and 37.0μg/mL, respectively. The lower IC_50_ value of the free radical scavenging compound, the stronger the antioxidant capacity. At their concentration of 1,600 mg/mL, the scavenging order of DPPH· was V_C_ > 1% (w/v)-ZOP-NPs-AgNPs > ZOP-NPs > 1% (w/v)-ZOP-AgNPs > ZOP > 0.5% (w/v)-ZOP-NPs-AgNPs > 0.5% (w/v)-ZOP-AgNPs. It could be seen that 1%-ZOP-NPs-AgNPs had high DPPH· radical scavenging ability. The IC_50_ values of 0.5% (w/v)-ZOP-AgNPs, 0.5% (w/v)-ZOP-NPs-AgNPs, 1% (w/v)-ZOP-AgNPs, 1% (w/v)-ZOP-NPs-AgNPs and Vc against OH· were 102.7, 74.8, 210.6, 243.5, and 16.8μg/mL, respectively. Similarly, 0.5%-ZOP-NPs-AgNPs showed better OH· scavenging ability. However, as the concentration increases, when the concentration reaches 1,600 mg/mL, the scavenging order of OH· radical was VC > 1% (w/v)-ZOP-NPs-AgNPs > 1% (w/v)-ZOP-AgNPs > 0.5% (w/v)-ZOP-NPs-AgNPs > 0.5% (w/v)-ZOP-AgNPs > ZOP-NPs > ZOP. Thus, the ability of several compounds to scavenge free radicals showed concentration dependence, which was consistent with the results of Cameron et al. (33).

In conclusion, ZOP mediated metal nanoparticles ZOP-NPs-AgNPs had a higher antioxidant activity on the formation of free radicals into the life system. Firstly, polysaccharides and their derivatives may play an antioxidant role through the interaction between electron transfer and hydrogen ions and free radicals (34). Second, ZOP-NPs-AgNPs had the characteristics of smaller particle size and larger specific surface area than other polysaccharide nanoparticles, which can integrate with more free radical scavenger and had a better antioxidant activity. Last but not least, the intermediate compounds formed by polysaccharide and metal ions during the formation of silver nanoparticles may also play a role in scavenging free radicals (35).

### Antimicrobial Activity

The antibacterial activities of ZOP-AgNPs and ZOP-NPs-AgNPs were studied in this paper, and the results are shown in Figures 9C,D. There was no inhibition zone around ZOP and ZOP-NPs, indicating that ZOP and ZOP-NPs had no antibacterial activity against *E. coli*. ZOP-AgNPs (0.5%, w/v; 1%, w/v) and ZOP-NPs-AgNPs (0.5%, w/v; 1%, w/v) had significant inhibitory effect on E. coli., and the size of inhibition zone of them were positively correlated with polysaccharide concentrations.

The diameters of their inhibition zones were 13.32, 13.67, 14.02, and 15.70mm, respectively. Wang et al. (36) showed that active silver nanoparticles may first destroy the cell membrane of bacteria, leading to the leakage of cell materials. The silver nanoparticles then enter the inner membrane of the cell and inactivate the respiratory chain dehydrogenase, thereby inhibiting the cell's respiration and growth. At the same time, silver particles can also affect the biological activity of some proteins and phosphates, causing the collapse of cell membranes, and ultimately leading to the decomposition and death of cells. For the silver nanoparticles, the larger the surface area, the more silver atoms attached to the solid surface, the stronger the antibacterial activity. Therefore, the antibacterial activity of silver nanoparticles is stronger than that of bulk silver (37). It could be seen from Figures 9C,D, with the increase of the concentration of ZOP-AgNPs and ZOP-NPs-AgNPs, their antibacterial activity also increased. In addition, ZOP-NPs-AgNPs had better antibacterial activity than ZOP-AgNPs at the same concentration. At different concentrations, with the increase of the concentration of AgNPs, that is, the number of polysaccharide nanoparticles in the same volume increases, more AgNPs can attack bacterial cells, resulting in the death of more bacterial cells, thus showing better antibacterial effect. In addition, ZOP-NPs-AgNPs had better antibacterial activity than ZOP-AgNPs at the same concentration. In other words, ZOP-NPs-AgNPs with small particle size showed higher antimicrobial activity.

In conclusion, under different concentrations, the antibacterial activity of AgNPs in a certain concentration range will increase with the increase of its concentration; Under the condition of the same concentration, the nanoparticles with smaller particle size, and larger specific surface area had better antibacterial performance.

## Conclusion

In this study, ZOP was used as raw material to prepare AgNPs. In this method, ZOP was used as a green reducing agent and stabilizer, overcoming the toxicity and pollution caused by chemical reducing agent in conventional methods. In conclusion, ZOP had the advantages of good reducibility and environmental protection, and provided a new method and approach for the green reduction of nano-silver. In addition, ZOP can also be used as a green reducing agent and stabilizer to further study its reducing and stability of other metals. Polysaccharide mediated generation of nano-silver, due to its good antibacterial activity, can be used in pharmaceutical, medical, food hygiene, preservation and other fields, to reduce microbial infection, and due to its high antioxidant activity, AgNPs will also be increasingly used in beauty products and the development of new antioxidants.

## Data Availability Statement

The original contributions presented in the study are included in the article/supplementary material, further inquiries can be directed to the corresponding author/s.

## Author Contributions

YJ and LW designed and conceived the study. YJ, WC, and YM performed the experiments. WC and YZ analyzed the data and drafted the manuscript. ML, YZ, and DZ contributed to the writing of the manuscript. YJ and DZ provided the funding and resources. All authors revised and approved the submitted version of the manuscript.

## Funding

This work was supported by the S & T Program of Hebei (No. H2021208007), the Key Research and Development Program of Hebei Province (No. 20370509D) and the Science and Technology Project of the Hebei Education Department (No. ZD2022021).

## Conflict of Interest

The authors declare that the research was conducted in the absence of any commercial or financial relationships that could be construed as a potential conflict of interest.

## Publisher's Note

All claims expressed in this article are solely those of the authors and do not necessarily represent those of their affiliated organizations, or those of the publisher, the editors and the reviewers. Any product that may be evaluated in this article, or claim that may be made by its manufacturer, is not guaranteed or endorsed by the publisher.
